# Impact of Cranioplasty Timing and Status on Long-Term Survival and Functional Outcomes After Decompressive Craniectomy for Severe Traumatic Brain Injury

**DOI:** 10.3390/brainsci15121336

**Published:** 2025-12-16

**Authors:** EJun Kim, Se Hyun Choi, Jee Hye Wee, Yi Hwa Choi, Hyuntaek Rim, In Bok Chang, Joon Ho Song, Yong-Kil Hong, Ji Hee Kim

**Affiliations:** 1 Department of Neurosurgery, College of Medicine, Hallym University, Anyang 14068, Republic of Korea; overken@naver.com (E.K.); coldwings@naver.com (H.R.); nscib71@hanmail.net (I.B.C.); song@hallym.or.kr (J.H.S.);; 2 Department of Ophthalmology, College of Medicine, Hallym University, Anyang 14068, Republic of Korea; choisehyun88@naver.com; 3 Department of Otorhinolaryngology-Head and Neck Surgery, College of Medicine, Hallym University, Anyang 14068, Republic of Korea; weejh07@gmail.com; 4 Department of Anesthesiology and Pain Medicine, College of Medicine, Hallym University, Anyang 14068, Republic of Korea; pcyhchoi@hallym.or.kr; 5 Department of Neurosurgery, Hallym University Sacred Heart Hospital, 22, Gwanpyeong-ro 170beon-gil, Dongan-gu, Anyang 14068, Republic of Korea

**Keywords:** cranioplasty, decompressive craniectomy, functional outcome, long-term survival, predictor, Rotterdam CT score, traumatic brain injury

## Abstract

**Background:** Decompressive craniectomy (DC) is a life-saving procedure for severe traumatic brain injury (TBI); however, its long-term outcomes remain controversial. Cranioplasty traditionally performed to restore cranial integrity, has been increasingly recognized for its potential role in neurological recovery. This study aimed to investigate the impact of cranioplasty timing and status on long-term mortality and functional outcomes after DC for severe TBI. **Methods:** We retrospectively reviewed 151 patients who underwent DC between 2014 and 2018. Patients were categorized into three groups according to cranioplasty timing: early (<3 months), late (≥3 months), and no cranioplasty. Clinical and radiologic data, including the Rotterdam CT scores, were analyzed. The primary endpoints were 5-year mortality and 12-month functional outcome assessed by the Glasgow Outcome Scale (GOS). Univariate and multivariate logistic regression analyses identified independent predictors and receiver operating characteristic (ROC) curves with are under the curve (AUC) values evaluated model performance. **Results:** Of 151 eligible patients (mean age = 53.9 ± 17.4 years; 82.1% male), overall 5-year mortality was 76.8% (116/151). Mortality differed substantially by cranioplasty group: 64.5% in early cranioplasty, 70.8% in late cranioplasty, and 82.3% in patients who did not undergo cranioplasty. Unfavorable 12-month functional outcomes occurred in 45.2%, 79.2%, and 91.7% of these groups, respectively. In multivariate analysis, no cranioplasty independently predicted both higher 5-year mortality (OR = 2.78, 95% CI = 1.06–7.25, *p* = 0.038) and unfavorable functional outcome (OR = 3.09, 95% CI = 1.18–8.09, *p* = 0.022). Older age was also associated with increased mortality (*p* = 0.019). ROC analysis showed moderate discriminative performance for 5-year mortality (AUC = 0.71) and good discrimination for unfavorable functional outcome (AUC = 0.80). **Conclusions:** Absence of cranioplasty was associated with higher long-term mortality and poorer functional recovery following DC for severe TBI. Early cranioplasty may enhance cerebral restoration and rehabilitation potential, improving both survival and neurological outcomes.

## 1. Introduction

Traumatic brain injury (TBI) remains a major global public health challenge and a leading cause of death and long-term disability across all age groups. Severe TBI frequently results in refractory intracranial hypertension, impaired cerebral perfusion, and progressive secondary brain injury, all of which critically influence neurological recovery. Decompressive craniectomy (DC), the surgical removal of a portion of a large bone flap with dural expansion, is widely performed as a life-saving surgical intervention to control medically refractory intracranial pressure, improve cerebral compliance, and restore cerebral oxygenation [1,2,3,4,5,6,7,8]. However, despite its physiological benefits, the long-term outcomes of DC continue to be debated, as many survivors experience substantial neurological disability, cognitive impairment, or delayed postoperative complications [9]. The two largest randomized controlled trials—DECRA and RESCUEicp—showed that although DC effectively lowers intracranial pressure, it may increase the proportion of survivors with severe disability at 6 months, raising questions about its long-term impact on functional recovery and health-related quality of life [10,11].

Radiological severity assessment using Rotterdam CT score, introduced by Maas et.al in 2005, has been widely adopted as an early prognostic indicator in severe TBI [12]. This scoring system integrates key CT findings—including basal cistern status, midline shift, traumatic subarachnoid hemorrhage (SAH) or intraventricular hemorrhage (IVH), and the presence of epidural hematoma (EDH)—to provide a simple, reproducible classification for early TBI evaluation [12] and demonstrates excellent interobserver reliability. Several studies have evaluated Rotterdam CT scores specifically in patients undergoing DC, showing that higher scores are associated with increased early mortality, unfavorable outcome, and postoperative complications [13,14,15,16]. However, the majority of these studies focused on in-hospital, 30-day, or 6–12 month outcomes, and the long-term prognostic value of the Rotterdam score beyond the first postoperative year remains unclear. Moreover, some studies have suggested persistent prognostic value over extended follow-up periods. Recent long-term cohort studies of severe TBI highlight that meaningful neurological recovery may continue for several years, even up to 5–8 years post-injury, and that survival curves and functional trajectories differ substantially from short-term outcome estimates [17,18]. Nevertheless, the Rotterdam CT score primarily reflects the initial structural severity of brain injury and does not capture the modifiable postoperative factors that may influence long-term neurorecovery.

Cranioplasty, traditionally viewed as a reconstructive procedure to restore cranial integrity after DC, has gained increasing attention for its physiological and neurological benefits. A growing body of evidence suggests that cranioplasty may improve cerebral blood flow, cerebrospinal fluid dynamics, metabolic activity, and neurocognitive function [19,20,21]. Clinical studies have reported improved rehabilitation engagement and functional recovery following cranioplasty, with some data supporting the benefits of early reconstruction performed within 3 months after DC [22,23]. However, cranioplasty timing varies widely across institutions, and its impact on long-term survival and functional outcomes remains insufficiently understood. Notably, most previous research has focused on short- or mid-term outcomes, leaving a significant knowledge gap regarding the influence of cranioplasty timing and performance on extended follow-up, particularly long-term survival beyond one year.

Given these uncertainties, a clearer understanding of whether cranioplasty timing or its omission affects long-term prognosis following DC is clinically important. Determining the extent to which cranioplasty serves as a modifiable determinant of long-term functional recovery and survival may provide actionable insights for surgical planning, rehabilitation strategies, and long-term patient counseling. Furthermore, integrating cranioplasty-related variables with established radiologic markers, such as the Rotterdam CT score, may help refine prognostic models for patients with severe TBI undergoing DC.

Therefore, the present study aimed to investigate the impact of cranioplasty timing and status on 12-month functional outcomes and 5-year mortality in patients who underwent DC for severe TBI. By combining clinical characteristics, radiological injury severity, and reconstructive surgical factors, this study seeks to clarify the long-term prognostic significance of cranioplasty within the broader context of postoperative neurorecovery after severe TBI.

## 2. Methods

### 2.1. Ethics

This retrospective single-center cohort study included consecutive adult patients who underwent DC for TBI between 2014 and 2018. The study protocol was approved by the Institutional Review Board (IRB) of Hallym University (IRB No. HALLYM 2019-05-032) and conducted in accordance with the principles of the Declaration of Helsinki. Because the investigation was based solely on a review of existing medical records, the requirement for informed consent was waived by the IRB.

### 2.2. Patient Population

**Figure 1:**
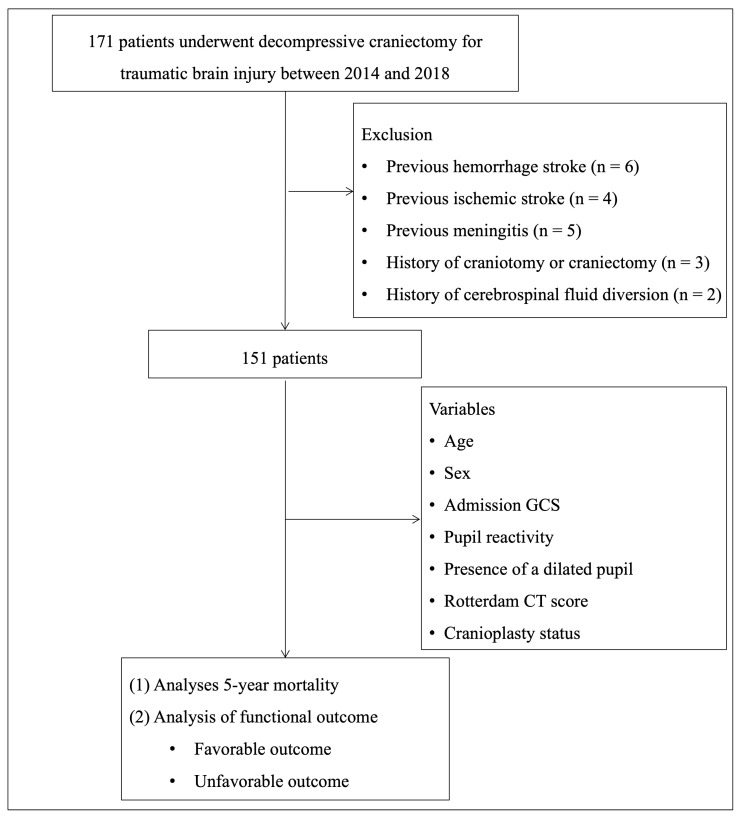
Flowchart of the patients screening process.

A total of 171 patients underwent DC for TBI during the study period. We excluded patients with previous hemorrhage stroke (n = 6) or ischemic stroke (n = 4), previous meningitis (n = 5), history of craniotomy or craniectomy (3), and prior cerebrospinal fluid (CSF) diversion procedures—such as ventriculoperitoneal shunt or endoscopic third ventriculostomy (n = 2). After applying these criteria, 151 patients were included in the final analysis. A study flow diagram illustrating patient inclusion and exclusion has been added as Figure 1. All operations were performed by four attending neurosurgeons using a standardized trauma flap technique. The indications for DC were based on the Brain Trauma Foundation guidelines for the management of intracranial pressure (ICP) after TBI, 4th edition [24]. Surgical criteria included an intracranial mass lesion with a midline shift greater than 10 mm, hematoma volume exceeding 30 mL (supratentorial) or 10 mL (infratentorial), or an admission Glasgow Coma Scale (GCS) score below 8. Patients with sustained ICP above 20 mmHg or progressive neurological deterioration were also candidates for surgery. In most cases, DC was accompanied by wide dural expansion using an artificial dural graft, and hematoma evacuation was performed when indicated. Postoperative therapeutic hypothermia was applied in cases of refractory brain swelling or excessive intraoperative brain bulging after DC.

### 2.3. Clinical Data Collection

Collected demographic and clinical data included age, sex, mechanism of injury, presence of major extracranial injuries, GCS at admission, as well as pupil size and reactivity. Treatment-related variables included type of DC, transient CSF drainage, postoperative hypothermia treatment, reoperation, and cranioplasty status, which was categorized into three groups based on surgical timing as follows: early cranioplasty (performed <3 months after DC), late cranioplasty (performed ≥3 months), and no cranioplasty (patients who never underwent the procedure).

### 2.4. Radiological Data Review

Preoperative CT findings were carefully reviewed to calculate the Rotterdam CT score by assessing major radiologic markers of traumatic injury. The evaluation included the presence of subarachnoid or intraventricular hemorrhage, subdural or epidural hematoma, intracerebral hemorrhage (ICH), the degree of midline shift, and the status of the basal cisterns. Each of these radiologic features carries a predefined numeric value according to the Rotterdam scoring system as follows: (1) midline shift (0 = no shift or ≤5 mm; 1 = shift > 5 mm), (2) epidural hemorrhage (0 = present, 1 = absent), (3) compression of basal cisterns (0 = normal, 1 = compressed, 2 = absent), (4) intraventricular blood or traumatic SAH (0 = absent, 1 = present). After assessing values to each component, the individual points are summed and an additional point is added to obtain the final Rotterdam CT score, which ranges from 1 to 6.

Postoperative CT images were reviewed to assess craniectomy size and the presence of postoperative findings including IVH, infarction, subdural hygroma, and hydrocephalus.

### 2.5. Outcome Assessment

The primary outcome was 5-year mortality, assessed using hospital medical records and the national death registry; thus, no loss to follow-up occurred for this endpoint. Functional outcome at 12 months after injury was evaluated using the Glasgow Outcome Scale (GOS), scored from 1 to 5 (1 = death, 2 = vegetative state, 3 = severe disability, 4 = moderate disability, 5 = good recovery), based on clinic visits. Patients who died within 12 months were assigned GOS 1. Unfavorable functional outcome was defined as GOS 1–3 and favorable outcome as GOS 4–5.

### 2.6. Statistical Analysis

Continuous variables were presented as mean ± standard deviation (SD) or median (interquartile range, IQR), and categorical variables as frequency and percentage. Group comparisons (survivors vs. non-survivors; favorable vs. unfavorable functional outcomes) were performed using Student’s *t*-test or Mann–Whitney U test for continuous variables and chi-square or Fisher’s exact test for categorical variables.

Variables included in multivariate logistic regression models were selected based on clinical relevance. The final models for 12-month unfavorable functional outcome and 5-year mortality included the following: age, sex, admission GCS, pupil reactivity, presence of a dilated pupil, Rotterdam CT score, and cranioplasty status.

To assess discrimination performance, receiver operating characteristic (ROC) curves were constructed for models predicting 5-year mortality using cranioplasty status, age, and Rotterdam CT score, as well as 12-month unfavorable functional outcome using all three cranioplasty categories (early, late, none), age, and Rotterdam CT score. The area under the curve (AUC) with 95% confidence intervals (CIs) was calculated for each model to evaluate predictive accuracy. The optimal cut-off value was determined using the Youden index (maximum [sensitivity + specificity − 1]). Corresponding sensitivity, specificity, positive predictive value (PPV), and negative predictive value (NPV) were also obtained. All statistical tests were two-tailed, with significance defined as a *p* value less than 0.05. Analyses were performed using SPSS version 22.0 (IBM Corp., Armonk, NY, USA).

## 3. Results

Of the 171 patients who underwent DC, 20 were excluded based on predetermined criteria, leaving 151 patients for the final cohort. The inclusion and exclusion process is summarized in Figure 1. A total of 151 patients who underwent DC for TBI were included in this study (Table 1). The mean age was 53.9 ± 17.4 years, and 124 patients (82.11%) were male. The most common mechanism of injury was motor vehicle accidents (47 patients, 31.12%), followed by falls (18 patients, 11.92%), slip down (38 patients, 25.16%), and other causes (48 patients, 31.78%). Major extracranial injuries were present in 34 patients (22.51%). At admission, 78 patients (51.65%) had a GCS score ≤ 6, and 79 patients (53.37%) had at least one dilated pupil. Pupillary light reaction was normal bilaterally in 66 patients (44.59%), unilateral in 16 patients (10.81%), and absent bilaterally in 66 patients (44.59%). Regarding the type of DC, unilateral craniectomy was performed in 133 patients (88.07%) followed by bilateral craniectomy (11 patients, 7.28%), bifrontal craniectomy (4 patients, 2.64%), and suboccipital craniectomy (3 patients, 1.98%). The mean craniectomy area was 414.92 ± 291.75 cm^3^. Postoperative CT findings revealed IVH in 56 patients (43.41%), cerebral infarction in 36 patients (27.90%), subdural hygroma in 30 patients (23.25%), and hydrocephalus in 19 patients (14.72%). Temporary CSF drainage was performed in 12 patients (7.94%), postoperative hypothermia in 10 patients (6.62%), and reoperation in 24 patients (15.89%). Cranioplasty was not performed in 96 patients (63.57%), performed within 3 months in 31 patients (20.59%), and after 3 months in 24 patients (15.89%). The distribution of Rotterdam CT scores was as follows: score 1 (0 patients), score 2 (2 patients, 1.32%), score 3 (8 patients, 5.29%), score 4 (42 patients, 27.81%), score 5 (57 patients, 37.74%), and score 6 (42 patients, 27.81%).

A total of 116 of 151 patients (76.82%) died within 5 years after DC. The 5-year mortality rates according to the Rotterdam CT score were 0%, 1.72%, 5.17%, 27.59%, 37.93%, and 27.59% for scores 1–6, respectively, showing a general trend of increasing mortality with higher scores. When stratified by cranioplasty status, the 5-year mortality rates were 17.24% in the early cranioplasty group, 14.66% in the late cranioplasty group, and 68.1% in the no-cranioplasty group. Both early and late cranioplasty groups showed substantially lower mortality compared with patients who never underwent cranioplasty. Similarly, the proportion of patients with unfavorable functional outcomes at 12 months (GOS 1–3) was lowest in the early cranioplasty group (45.16%), followed by late (79.17%) and no cranioplasty (91.67%) groups (Table 2).

In the binary multivariate logistic regression model (Table 3), no cranioplasty emerged as an independent predictor of 12-month unfavorable functional outcome (adjusted odds ratio [OR] = 3.09, 95% confidence interval [CI] = 1.18–8.09, *p* = 0.022). Age was also associated with worse outcome (OR = 1.03, 95% CI = 1.00–1.07, *p* = 0.046). Other variables—including sex, GCS at admission, Rotterdam CT score, pupil reactivity, pupil dilation, and late cranioplasty—were not statistically significant predictors.

Multivariate logistic regression identified no cranioplasty as a significant independent risk factor for 5-year mortality (OR = 2.78, 95% CI = 1.06–7.25, *p* = 0.038). Older age was also associated with increased mortality (OR = 1.04, 95% CI 1.01–1.07, *p* = 0.019). Late cranioplasty, GCS, Rotterdam CT score, and pupillary findings did not reach statistical significance (Table 4).

ROC curve analysis demonstrated acceptable discriminatory performance for both predictive models (Supplementary Figure S1). For 5-year mortality, the combined model incorporating cranioplasty status (binary: performed vs. not performed)), age, and Rotterdam CT score yielded an AUROC of 0.71 (95% CI, 0.62–0.80), with 78% sensitivity and 63% specificity at the optimal cut-off (0.46). Based on the observed 5-year mortality rate in our cohort, the model showed a positive predictive value (PPV) of 87.5% and a negative predictive value (NPV) of 46.4%. For 12-month unfavorable functional outcome, the model including the three cranioplasty categories (early, late, and none) together with age and Rotterdam CT score achieved an AUROC of 0.80 (95% CI, 0.72–0.88), with 81% sensitivity and 69% specificity at the optimal cut-off (0.52). Considering the prevalence of unfavorable outcomes in the study population, this model demonstrated a PPV of 91.3% and an NPV of 47.4%.

## 4. Discussion

This study demonstrated that the absence of cranioplasty following DC was independently associated with higher 5-year mortality and poorer 12-month functional outcomes in patients with severe TBI. In contrast, patients who underwent early cranioplasty (<3 months) exhibited more favorable long-term survival and neurological recovery. These findings support the view that cranioplasty should not be regarded merely as a reconstructive procedure but as an integral component of neurorestorative management after DC.

The long-term prognosis after severe TBI remains highly variable. Systematic reviews report that 18–75% of patients die within several years of injury, and less than one-third regain functional independence beyond 2 years [25]. Consistent with prior large-scale studies, our cohort showed a 5-year mortality rate of 64.9%. Advanced age and greater initial neurological severity—reflected by low GCS and high Rotterdam CT scores—were also linked to poor outcomes, confirming their well-established prognostic significance. However, our findings expand these models by showing that postoperative reconstructive and rehabilitative factors—especially cranioplasty status—significantly modify the long-term trajectory beyond the initial injury severity.

The optimal timing of cranioplasty has long been debated. The multicenter CENTER-TBI and Net-QuRe study by Vreeburg et al. found no significant difference in 12-month functional outcome between early (≤90 days) and delayed (>90 days) cranioplasty, although early surgery was associated with a slightly higher incidence of hydrocephalus [26]. Similarly, Aloraidi et al. reported no significant difference in GOS or modified Rankin Scale between early and late cranioplasty, suggesting that timing should be individualized according to cerebral edema resolution [27]. In contrast, a meta-analysis by De Cola et al. demonstrated that cranioplasty performed within 3 months of DC improved motor and cognitive recovery without increasing complications. Likewise, Beauchamp et al. emphasized that timely restoration of cranial integrity may favorably influence cerebral physiology and long-term neurological restoration [28].

Our results partially align with these observations. While early and late cranioplasty groups achieved comparable short-term outcomes, patients without cranioplasty exhibited markedly poorer long-term survival and functional recovery. This suggests that although the exact timing may be flexible, performing cranioplasty itself—preferably after resolution of brain swelling—remains crucial for optimizing recovery. Restoration of skull integrity improves cortical metabolism and perfusion, which may facilitate neuroplasticity and rehabilitation efficacy.

Physiologically, cranioplasty normalizes ICP gradients, enhances venous return, and restores CSF circulation. Perfusion and metabolic imaging have demonstrated improved regional blood flow and neural activity following cranioplasty, paralleling cognitive and motor recovery—a reversal of the “syndrome of the trephined” [25]. Early reconstruction also enables earlier engagement in rehabilitation and cognitive therapy, accelerating functional reintegration [29]. Thus, the neurophysiological benefits of cranioplasty extend well beyond cosmetic repair, constituting a neurorestorative intervention that actively shapes recovery trajectories [30]. Overall, cranioplasty restores normal ICP gradients, improves cerebral blood flow and metabolism, normalizes CSF circulation, and enhances venous outflow. These physiological improvements may reverse the syndrome of the trephined and facilitate more effective participation in rehabilitation. These mechanisms support the beneficial association observed between cranioplasty and long-term outcomes in our cohort.

The Rotterdam CT score remains a robust early prognostic marker for TBI severity [31,32]. Yet, in our multivariate analysis, its long-term predictive power diminished once cranioplasty status was incorporated, suggesting that secondary interventions and rehabilitation can modulate recovery beyond initial injury severity. To isolate the independent contribution of cranioplasty, our models adjusted for Rotterdam score, age, GCS, and pupil reactivity. After controlling for these confounders, cranioplasty status persisted as a strong independent predictor of both 5-year survival and 12-month functional recovery. As illustrated in Figure 1, incorporating cranioplasty status into the prognostic model alongside age and Rotterdam CT score significantly enhanced predictive discrimination (AUC = 0.71 for 5-year mortality; AUC = 0.80 for 12-month unfavorable functional outcome). Sensitivity and specificity at optimal threshold were 78%/63% and 81%/69%, respectively, indicating good model performance. Given the high prevalence of adverse outcomes in this cohort, the predictive models demonstrated strong rule-in capability, with PPVs of 87.5% for 5-year mortality and 91.3% for 12-month unfavorable functional outcome. However, the relatively low NPVs (46.4% and 47.4%, respectively) indicate limited ability to confidently excluded poor outcomes, suggesting that the models are more suitable for identifying high-risk patients rather than reliably ruling out low-risk cases. These findings emphasize that while the Rotterdam CT score reflects early structural injury, postoperative reconstructive variables such as cranioplasty and substantial prognostic value for long-term outcomes. Collectively, our study underscores that cranioplasty is a modifiable, quantifiable determinant of long-term prognosis following DC for severe TBI. Integrating surgical timing and cranioplasty status into conventional prognostic models may refine individualized risk prediction, facilitate early rehabilitation planning, and guide long-term management strategies in TBI care.

Meanwhile, the proportion of female patients in our cohort was relatively low, which is consistent with the epidemiology of severe TBI. Males are known to have a substantially higher incidence of high-energy trauma due to greater exposure to traffic accidents, occupational hazards, and risk-taking behaviors, and this demographic pattern was reflected in our study population.

## 5. Limitations

Our predictive models have several limitations. First, not all components of validated prognostic scoring systems such as the IMPACT and CRASH models were available due to the retrospective design, which may reduce the completeness of risk adjustment. Second, although we used multivariable analysis, unmeasured confounders—particularly those influencing the decision to perform cranioplasty—may persist. Patients who did not undergo cranioplasty generally had more severe neurological damage, medical instability, or complications such as infections or refractory hydrocephalus (Supplementary Table S1). This introduces confounding by indication, as poorer outcomes may be driven by initial severity rather than the absence of cranioplasty. Third, the sample size limited the number of variables that could be included in the models without overfitting. Fourth, because this was a single-center study, institutional practices may limit the generalizability of our findings. Fifth, the Rotterdam CT score reflects only the initial structural injury and does not capture secondary insults, postoperative complications, or dynamic changes during intensive care. Sixth, selection bias, non-randomized timing of cranioplasty, and potential loss to follow-up cannot be excluded. Functional outcomes were assessed using the GOS, which lacks granularity for cognitive or psychosocial recovery. Lastly, causality between cranioplasty and survival cannot be definitively established. Therefore, the sensitivity and specificity of the models should be interpreted with caution.

Despite these limitations, our study demonstrates that the cranioplasty status and timing are a robust and independent predictors of both 12-month functional outcome and 5-year survival in severe TBI patients undergoing DC. These findings highlight the diagnostic and prognostic value of early cranioplasty and support the integration of cranial reconstruction into long-term outcome prediction and clinical decision-making. The sensitivity and specificity of the current models could be improved in future work by incorporating additional prognostic variables that were unavailable in this retrospective dataset, such as detailed physiological parameters, laboratory variables, intraoperative findings, and rehabilitation intensity. Machine-learning-based methods or external validation using multi-center datasets may also enhance predictive accuracy and generalizability.

## 6. Conclusions

In summary, this study highlights that cranioplasty status and timing are significant, modifiable determinants of long-term prognosis after DC for severe TBI. Early or timely restoration of cranial integrity may facilitate cerebral recovery, enhance rehabilitation potential, and improve both survival and functional outcomes. Incorporating cranioplasty variables into prognostic models alongside clinical and radiological factors such as the Rotterdam CT score may refine individualized risk prediction and guide long-term management in patients with severe TBI.

## Figures and Tables

**Table 1:** Summary of clinical characteristics.

Characteristics		Value
Age in years (mean ± SD)		53.89 ± 17.41
Male sex		124 (82.11)
Mechanism of injury		
	Motor vehicle accident	47 (31.12)
	Fall from a height	18 (11.92)
	Slip down	38 (25.16)
	Others	48 (31.78)
Major extracranial injury		34 (22.51)
GCS at admission ≤ 6		78 (51.65)
Pupils react to light ^†^		
	Both	66 (44.59)
	One	16 (10.81)
	None	66 (44.59)
At least 1 dilated pupil ^†^		79 (53.37)
Type of decompressive craniectomy		
	Unilateral craniectomy	133 (88.07)
	Bilateral craniectomy	11 (7.28)
	Bifrontal craniectomy	4 (2.64)
	Suboccipital craniectomy	3 (1.98)
Craniectomy size (cm^3^) (mean ± SD)		414.92 ± 291.75
Postoperative CT findings ^‡^		
	IVH	56 (43.41)
	Infarction	36 (27.90)
	Subdural hygroma	30 (23.25)
	Hydrocephalus	19 (14.72)
Transient CSF drain		12 (7.94)
Postoperative hypothermia		10 (6.62)
Reoperation		24 (15.89)
Cranioplasty		
	None	96 (63.57)
	Within 3 months	31 (20.59)
	After 3 months	24 (15.89)
Rotterdam score (/6)		
	1	0
	2	2 (1.32)
	3	8 (5.29)
	4	42 (27.81)
	5	57 (37.74)
	6	42 (27.81)

CSF, cerebrospinal fluid; GCS, Glasgow coma scale; IVH, intraventricular hemorrhage; SAH, subarachnoid hemorrhage; SD, standard deviation. All values are represented as the numbers of patients (% of total) unless otherwise indicated. ^†^ Missing data were included. Total 148 patients were analyzed except for 3 patients experiencing eye surgery. ^‡^ Missing data were included. Total 129 patients were analyzed except for 21 patients who were able to perform postoperative CT scan.

**Table 2:** Relationships of long-term outcomes with Rotterdam CT scores and cranioplasty groups.

Variables		No. of Patients (%)	Patients Who Died at 5 Years (/116)	Patients with Unfavorable Outcome at 12 Months (/121)
**Rotterdam CT score**				
	1	0	0	0
	2	2 (1.32)	2 (1.72)	0
	3	8 (5.30)	6 (5.17)	6 (75)
	4	42 (27.81)	32 (27.59)	33 (78.57)
	5	57 (37.75)	44 (37.93)	48 (84.21)
	6	42 (27.81)	32 (27.59)	34 (80.95)
**Cranioplasty group**				
	Early cranioplasty	31	20 (17.24)	14 (45.16)
	Late cranioplasty	24	17 (14.66)	19 (79.17)
	No cranioplasty	96	79 (68.1)	88 (91.67)

All values are represented as the numbers of patients (% of total) unless otherwise indicated.

**Table 3:** Binary multivariate logistic analysis to identify factors predicting 12-month unfavorable functional outcome.

Factors	Adjusted Odds Ratio	95% Confidence Interval	*p* Value
Age	1.03	1.00–1.066	0.046 *
Male sex	0.87	0.38–1.97	0.735
GCS	0.89	0.75–1.06	0.194
Pupils react to light	0.92	0.55–1.52	0.743
At least 1 dilated pupil	1.28	0.76–2.17	0.349
Late cranioplasty (≥3 months)	1.21	0.45–3.28	0.703
No cranioplasty	3.09	1.18–8.09	0.022 *
Rotterdam CT score	1.11	0.74–1.66	0.609

GCS, Glasgow coma scale. * Significance at *p* < 0.05.

**Table 4:** Binary multivariate logistic analysis to predict factors for 5-year mortality.

Factors	Adjusted Odds Ratio	95% Confidence Interval	*p* Value
Age	1.04	1.01–1.07	0.019 *
Male sex	0.93	0.39–2.20	0.871
GCS	0.86	0.73–1.02	0.081
Pupils react to light	0.97	0.59–1.59	0.913
At least 1 dilated pupil	1.19	0.69–2.05	0.528
Late cranioplasty (≥3 months)	1.18	0.37–3.78	0.780
No cranioplasty	2.78	1.06–7.25	0.038 *
Rotterdam CT score	1.17	0.69–2.05	0.528

GCS, Glasgow coma scale. * Significance at *p* < 0.05.

## Data Availability

The data presented in this study are available on request from the corresponding author (kimjihee.ns@gmail.com) due to privacy/ethical restrictions.

## Supplementary Materials

The following supporting information can be downloaded at: https://www.mdpi.com/article/10.3390/brainsci15121336/s1. Figure S1. Receiver operating characteristic (ROC) curves demonstrating the predictive performance of continuous variables significantly associated with long-term outcomes following decompressive craniectomy for severe traumatic brain injury. Table S1. Baseline characteristics according to cranioplasty status.
