# Supplementary material for: Impact of Cranioplasty Timing and Status on Long-Term Survival and Functional Outcomes After Decompressive Craniectomy for Severe Traumatic Brain Injury
Source: Brain Sci. 2025 Dec 16;15(12):1336. doi: 10.3390/brainsci15121336 (PMC12730317; doi:10.3390/brainsci15121336)
Supplement: Supplementary file 1 [file brainsci-15-01336-s001.zip › brainsci-4011761-supplementary.pdf]

Supplementary material

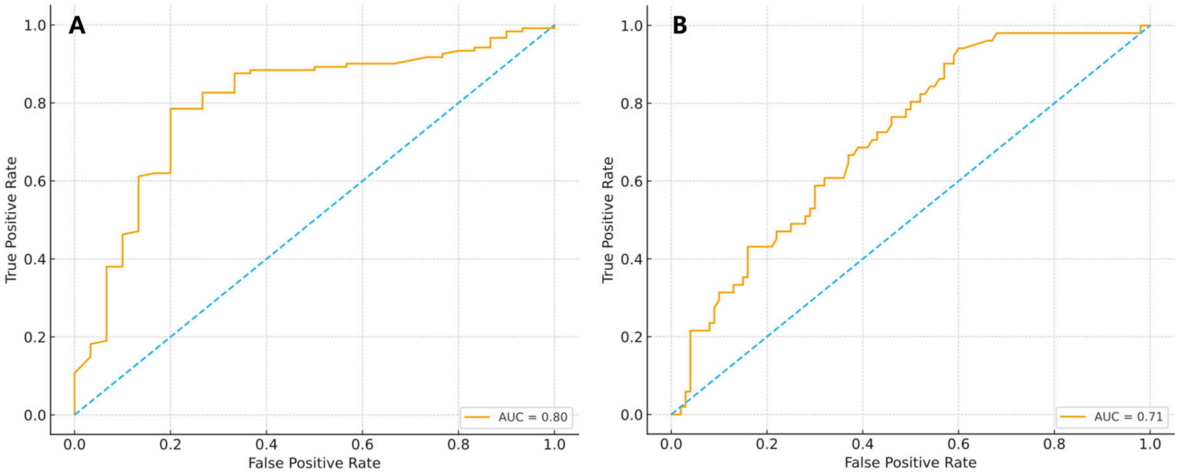

Figure S1. Receiver operating characteristic (ROC) curves demonstrating the predictive performance of continuous variables significantly associated with long-term outcomes following decompressive craniectomy for severe traumatic brain injury. ROC curves are shown for age, admission Glasgow Coma Scale (GCS), presence of at least one dilated pupil, and Rotterdam CT score. The area under the curve (AUC) values indicate the discriminatory ability of each variable for predicting (A) 12-month unfavorable functional outcome and (B) 5-year mortality.

Table S1. Baseline characteristics according to cranioplasty status

**Characteristics**<br>**No. of patients**	**Early cranioplasty (n = 31)**	**Late cranioplasty (n = 24)**	**No cranioplasty (n = 96)**
Mean age (years)	51	54	55
Male sex	29	16	80
Mechanism of injury			
Motor vehicle accident	10	10	28
Fall from a height	5	0	13
Slip down	9	9	21
Others	7	8	34
Major extracranial injury	5	5	24
GCS at admission ≤6	8	11	58
Pupils react to light†			
Both	21	15	31

One	6	2	8
None	4	7	55
At least 1 dilated pupil†	9	10	60
Type of decompressive craniectomy			
Unilateral craniectomy	28	22	83
Bilateral craniectomy	2	2	7
Bifrontal craniectomy	1	0	4
Suboccipital craniectomy	0	0	2
Mean craniectomy size (cm^3)	472.6	387.7	399.8
Postoperative CT findings‡			
IVH	7	9	40
Infarction	0	2	34
Subdural hygroma	5	6	19
Hydrocephalus	4	6	9
Transient CSF drain	4	1	7
Postoperative hypothermia	2	3	5
Reoperation	3	6	20
Rotterdam score (/6)			
1	0	0	2
2	3	2	3
3	6	9	27
4	16	11	30
5	6	2	34
6	0	0	0
